# Implementation and development of hospital-based health technology assessment in Poland from the perspective of hospital representatives: qualitative research

**DOI:** 10.3389/fpubh.2024.1426420

**Published:** 2024-10-08

**Authors:** Maciej Furman, Małgorzata Gałązka-Sobotka, Iwona Kowalska-Bobko

**Affiliations:** ^1^Institute of Public Health, Health Policy and Management Department, Health Sciences Faculty, Jagiellonian University Medical College, Cracow, Poland; ^2^Institute of Healthcare Management, Łazarski University, Warsaw, Poland

**Keywords:** HB-HTA, in-depth interviews, qualitative study, hospitals, medical technologies

## Abstract

**Introduction:**

The analysis of health technologies in Poland has so far mainly concerned drugs assessed by the Agency for Health Technology Assessment and Tariffication, which was established for this purpose. Due to the implementation of various forms of investment (hardware, diagnostic, and organizational) in hospitals, and the growing need to properly assess the costs of implemented solutions, Hospital-Based Health Technology Assessment (HB-HTA) began to develop. In order to implement HB-HTA in the Polish healthcare system, in 2019–2022 a project was carried out, the consortium members of which were: the National Health Fund (NHF), the National Institute of Cardiology in Warsaw (NIKARD), and Lazarski University (UŁ).

**Purpose of the study:**

Analyzing the possibilities of developing HB-HTA in Polish hospitals based on the experiences of the study participants. Presenting benefits and barriers for HB-HTA units implementation to Polish hospitals.

**Methods:**

Qualitative analysis of in-depth interviews with the representatives of hospitals that participated in the HB-HTA project using the Nvivo12 program. A specially prepared questionnaire with questions regarding the pre-project, design, and post-project phases was presented to respondents from five of the seven hospitals participating in the pilot phase of the project, during which HB-HTA reports were prepared for selected medical technologies. The remaining two facilities that were invited refused to participate in the study. The Ethics Committee of the Jagiellonian University Medical College gave consent to conduct the study.

**Results:**

Hospital representatives indicate that the HB-HTA methodology allows for the assessment of investments in facilities based on appropriate data. Thanks to the project, employees of hospital units gained new skills, such as becoming familiar with literature reviews in medical bibliographic databases. However, HB-HTA in Poland has not been fully implemented because facilities do not obtain adequate benefits from the implementation of HB-HTA at the organizational and financial level. According to the study participants, the methodology itself should be modified to take into account the needs of the facility.

**Conclusion:**

The hospitals participating in the HB-HTA project are developing the field of analyzing how innovative solutions are implemented in their facilities, but not to the extent that was intended in the project design.

## 1 Introduction

Health technology assessment primarily refers to justifiying the inclusion of health services or drug technologies to a list of services reimbursed by the public insurer. In Poland, there is a special advisory institution that helps the Minister of Health make decisions about financing a given solution from public funds, the Agency for Health Technology Assessment and Tariffication (*pol. Agencja Oceny Technologii Medycznych i Taryfikacji,* AOTMiT). The Agency’s activities are based on the Act on Healthcare Services Financed From Public Funds (1). It also deals with the tariffication of health services and the evaluation of health policy programs. This agency cooperates with various entities in the healthcare system, including hospitals, regarding the transmission of relevant data, among other things (2). This institution has been operating since 2005 and has an established position in the healthcare system. However, Poland lacks both regulations and long-term experience in assessing therapeutic and diagnostic technologies at the hospital level. The data from hospitals were not taken into account in the process of evaluating medical services and including medical innovations in the guaranteed services package funded by the public payer. The role of hospitals in shaping HTA was limited, and the data from hospitals did not play that role as they could have. Hospitals suffered from low financing of medical services evaluated by AOTMiT, and many of them experienced difficulties with cash flow and financial instability.

Changes related to the introduction of modern medical technologies at the hospital level require the participation of many actors in the healthcare system. There is a noticeable need among Polish healthcare facilities to develop various technologies, such as da Vinci or Versius robots. In particular, 2022 was a breakthrough year for the development of medical robotics, with the number of surgical robots increasing significantly. There have also been trends for some large centers, both public and private, to increase the number of procedures using medical robots (3). The number of procedures performed in public facilities exceeded the number of procedures performed in private facilities. At the same time, the number of commercial robotic procedures financed privately by patients decreased from approximately 1,225 in 2021 (16% of the total) to 900 in 2022 (9%), the vast majority of which (69%) were radical prostatectomies (4). The market for private surgeries (mainly prostatectomies) shrank primarily due to a significant increase in National Health Fund valuations, thanks to which several large private hospitals signed contracts with the fund and began to offer the procedures free of charge to patients (5). The use of medical robots could be an area of interest for people involved in introducing modern treatment techniques or equipment to facilitate diagnosis and ensure the development of medical facilities. Such technologies could be evaluated in accordance with the HB-HTA methodology. The provision of data by hospitals would facilitate more expedient decision-making by stakeholders with regard to the financing of such innovations. HB-HTA represents a methodology for the dissemination of new technologies.

Modern technologies create trends in hospitals because, thanks to them, hospital administrators can set their priorities and long-term plans. Other bodies related to healthcare are also involved in the process of creating those plans, for example the Ministry of Health, which has created its own department for the development of new technologies (Department of Innovations) (6). This department is responsible for managing e-health projects and supervising the Medical Research Agency (liable for non-commercial clinical trials) and the E-Health Center. The Department of Innovations is engaged in planning and coordinating the implementation of new technologies in the fields of health and prevention, as well as preparing guidelines for the implementation of the National Economy Reconstruction Plan after the Covid-19 pandemic in the area of the digitization of healthcare and the digital security of entities performing medical activities (7).

Evaluation Instrument of Investment Motions in Health Care (pol. *Instrument Oceny Wniosków Inwestycyjnych w Sektorze Zdrowotnym*, IOWISZ) plays a significant role in organizing investments in hospital facilities in Poland. It is a tool for voivodes and the Minister of Health to evaluate the advisability of investments. It was introduced to ensure that new investments are made where they are most needed and where they will bring the greatest benefits to patients (8).

The main goal of the IOWISZ system was to properly supervise and organize the investment process in healthcare, while developing an effective and fully rational system of spending public funds. One may use the IOWISZ IT system to submit a request for an opinion on the role of a given investment and its purpose. At the same time, it is used by the Minister of Health and voivodes to evaluate applications and issue opinions after consulting the President or Directors of the National Health Fund, respectively. An intention of the system was to enable service providers to develop prospectively, in line with local health needs, after obtaining a positive opinion from individual stakeholders of the system (9).

Support for implementing innovative medical technologies in hospitals and enriching the IOWISZ methodology was to be provided by the Hospital Assessment of Innovative Health Technologies (HB-HTA) project, which was carried out by three consortium members: the National Health Fund (NFZ), the National Institute of Cardiology in Warsaw (NIKARD), and Lazarski University in Warsaw (UŁ). It was financed by the National Center for Research and Development and was implemented during the years 2019–2022. The project was divided into two design phases: research and implementation (10).

As part of the project research design stage, six HB-HTA implementation theoretical models were prepared (11–16). These models were developed based on surveys with hospitals regarding innovative medical technologies introduction, surveys with patients from cardiology and oncology department, ethical aspects analysis related to innovative medical technologies introducing, literature review about HB-HTA network in Europe, a systematic analysis of reports from hospital health technology assessment units, an analysis of a functional benchmark which compared the practice of health technology assessment in Polish hospitals to an example HB-HTA process, which was described on the basis of a review of international practices, interviews with hospital management staff on the possibility of introducing HB-HTA in their facilities (17, 18).

Each theoretical model for the implementation of HB-HTA in Poland was developed according to the same scheme. Models were analyzed based on identification of stakeholders, key entities in the HB-HTA process, stakeholder analysis of supporting, inhibiting forces and neutral forces, analysis of the interests of individual stakeholders and the consequences of their actions, analysis of the relationship between stakeholders — structural model, the course of the HB-HTA process, the relationship of entities in the context of the flow of data, products and services within the model, analysis of the competence potential of the coordinating unit, financial aspects and the possibility of maintaining the network, analysis of barriers and opportunities for the analyzed scenario and summary, differentiating criteria (19).

Based on the theoretical models and assumptions, the roles of the various healthcare institutions that are to participate in the creation of the HB-HTA network in Poland was indicated. They included hospitals themselves, AOTMiT, the National Health Fund, the Ministry of Health, regional authorities, and external HTA units. In the pilot phase of the project, selected hospitals were obliged to prepare HB-HTA reports in accordance with the specific methodology prepared in the project.

Seven hospitals renowned for their notable contributions to medical research and their expertise in implementing innovative medical technologies, which had past engagements in projects organized by Łazarski University, were selected to participate. These hospitals comprised the Military Medical Institute in Warsaw, the University Medical Center in Gdańsk, the Lower Silesian Cancer Center in Wrocław, the University Hospital in Kraków, the Central Clinical Hospital of the Medical University of Łódz, the Center of Child Health in Warsaw, and the National Institute of Cardiology in Warsaw (20).

The personnel within these hospitals underwent intensive training in HB-HTA, facilitated by the project consortium. This training capitalized on the knowledge and materials generated in the previous phases of the project. Subsequently, pilot HB-HTA units were established within these hospitals and were tasked with conducting pilot assessments of selected innovative medical technologies and producing HB-HTA reports for these technologies. The assessed technologies encompassed the integration of the Da Vinci robotic surgery system; the establishment of an integrated care center for advanced Chronic Obstructive Pulmonary Disease (COPD) patients; enhancements to the organization and processes related to cervical cancer diagnosis; a comparative evaluation of implantable electrocardiogram (ECG) recorders in contrast to traditional ECG and Holter diagnostics; an evaluation of bipolar ablation in patients with cardiac arrhythmias; an assessment of contemporary diagnostic and therapeutic procedures for retinoblastoma management in children (inclusive of the role of intra-arterial selective chemotherapy in the overall treatment paradigm); and an evaluation of the impact of myocardial perfusion using multi-slice computed tomography, complemented by alternative communication techniques, within the context of ischemic heart patients (21).

The aim of this study is to indicate how HB-HTA should be further developed in Poland based on the analysis IDI (in-depth interviews) with representatives of five out of the seven hospitals that participated in the pilot phase of the HB-HTA project and identify the obstacles and opportunities for further HB-HTA implementation at the level of Polish hospitals. Thanks to that it would be easier to provide answers to questions regarding the development and introduction of innovations in medical entities using HB-HTA methodology in Poland.

## 2 Methods

### 2.1 Introduction to the study

Five hospitals that were involved in preparing HB-HTA reports decided to participate in the study. The remaining two entities (the Children’s Health Center in Warsaw and the Military Medical Institute in Warsaw) refused to participate due to having no further HB-HTA activities. A total of five participants took part in the study, all from facilities involved in the pilot phase of HB-HTA. Before the respondents agreed to participate, they had been sent a letter electronically, which was describing the purpose of the study and the topics that would be discussed during the interview. The remaining section of the invitation was distributed via email, which included a detailed description of the study’s objective and the proposed interview structure. The list was distributed to the hospital’s senior management. The participants for the interview were selected based on the decisions of the hospital managers and their engagement with the HB-HTA project. Informed consent forms were sent to all study participants, which they signed and returned to the interviewing researcher (MF). The participants of the study were involved in the preparation of HB-HTA reports between October and December 2021 and presented that reports during Polish HB-HTA conference in February 2022. Furthermore, they had participated in training courses prepared by Lazarski University and were identified as leaders within their respective entities within HB-HTA. The selection was based on the candidates’ previous experience with the HB-HTA project and their potential to establish HB-HTA groups of specialists. The hospitals were selected on the basis of an analysis of the networks of HB-HTA in Western Europe. The participants in the study were also selected based on their experience with HB-HTA and their contact with one of the project consortium partners, Lazarski University.

### 2.2 Designing the study

A qualitative in-depth interview study was conducted with a group of hospital representatives. The interviews were recorded, then transcribed and translated into English (conversation transcripts can be found in Supplementary materials (Supplementary Data Sheets 1-5)). The set of questions was presented to the respondents prior to the interviews so they could familiarize themselves with the presented topic. The scenario for interviews was prepared by two members of the research team (IKB and MF) and divided into three, parts due to the nature of the questions asked (pre-project phase, design phase, post-project phase). The interviews were conducted between February and March 2024 in the form of an online teleconference via MS Teams. Each interview lasted from 20 to 30 min, during which questions were asked according to a previously developed scenario (Table 1). Each interviewee responded to the same set of questions, posed by the researcher (MF), in a single session. This approach was adopted to ensure comparability across all interviews for the purposes of analysis. All interviewees were afforded the opportunity to respond to the questions in a manner of their choosing, without any restrictions as to the form of their responses. In conclusion, five individual interviews were conducted as part of the study. It was not possible for respondents to participate in the same sessions.

**Table 1:** Study group characteristics.

Study participant number	Gender	Name of entity	Position of the facility representative
P1	Male	University Clinical Center in Gdansk	Head of the Innovation, Analytics, and Medical Technology Implementation Department
P2	Male	Lower Silesian Oncology Center in Wroclaw	Head of the Department of Oncological Strategy and Profilaxis
P3	Male	National Institute of Cardiology in Warsaw	Director’s Representative for Innovative Business Models
P4	Female	Central Clinical Hospital of the Medical University of Lodz	Employee of the Organizational Department
P5	Male	University Hospital in Krakow	Financial Audit and Control Manager

Source: author’s own research.

The following research was prepared through internal discussion between the research team (IKB, MGS and MF) regarding the most suitable questions for exploring the implementation of HB-HTA in Polish hospitals. Of the seven selected hospitals invited to participate in the study, five representatives took part in the research. From the aforementioned seven hospitals, five were selected for inclusion in the study.

### 2.3 Ethical issues

The study was anonymized, and the information provided describes only the employee’s function and the institution they come from. Each interview was labeled with a number from 1 to 5. Consent to conduct the study was obtained by submitting an application to the Research Ethics Committee of the Jagiellonian University Medical College (opinion no. 118.6120.146.2023).

### 2.4 Data analysis

Content analysis (thematic analysis) was performed using the Nvivo12 program. A coding method was used based on description of the presented positions (*Description focused-coding*). The content of the interviews was analyzed by two members of the research team (IKB and MF). The concepts were identified and categorised based on the content of the interviewees’ responses and related to questions included into scenario. Subsequently, the researchers proceeded to code for the frequency of the concept and ascertain the number of times the code was repeated in the interviews of all respondents. The text was then subjected to a process of coding, whereby the data was divided into subcodes. Ultimately, five overarching concepts, comprising eleven codes and ten subcodes, were identified and subjected to analysis. The results are presented in Table 2.

**Table 2:** Concepts, codes and subcodes from analyzed interviews with hospital representatives.

Concepts	Codes	Subcodes
Information about the project and reasons for participating in it	Management support Healthcare service valuation New technologies evaluation	Managers initiatives Better cost estimation Appropriate methodology
Methods of assessing health technologies before the project	Internal analyses and IOWISZ, external institutions	External institutions Troubles with IOWISZ
Benefits resulting from participation in the study	Systematic way to analyse medical technologies	EBM and medical knowledge
Organizational and financial changes to implement HB-HTA in hospitals and the healthcare system	Financial factor New standards in data processing Maintaining the team Problems with benefits for hospitals	Lack of engagement Lack of financing
The need to modify the HB-HTA methodology	HB-HTA as difficult methodology	Document shortening Crucial information report Methodology simplification

## 3 Results

### 3.1 Information about the project and reasons for participating in it

Respondents indicated that they had learned about the HB-HTA project from various sources. One of the respondents (P3) was responsible for organizing the project itself at the beginning of its operation as one of the consortium members. It shows that hospital entities were one of facilitators for introducing HB-HTA at the level of Polish healthcare system: “We were the initiators of the idea itself, so this question does not directly apply to us” (P3). In Poland, medical facilities were one of the initiators of implementing HB-HTA into the Polish healthcare system.

#### 3.1.1 Management support

The remaining participants indicated various websites and facility directors (involved in the initial phases of the HB-HTA project) as the main sources of information about the project. “I found the information on the Internet, but our director and the director of the Institute of Cardiology also exchanged information” (P2). Directors and management boards were interested in finding information about the project and were initiators of actions. They were active in getting to know the project’s goals: “On the *GrupaBiznes* website I found a document with an invitation to the HB-HTA pilot project and our director also got interested in the project” (P4). “I am not sure exactly how the hospital found out about the project, but the HB-HTA team was formed by the contemporary director” (P5). Among the reasons for participating in the project, the respondents mentioned the nature of their facilities, which are drawn to new projects, as they regularly implement innovative medical technologies: “We are an innovative institution, and we place great emphasis on that field. We try to take part in multiple projects” (P1). The facilities selected for the study showed great willingness to implement innovations in various fields of medicine. Respondents also indicated that basing decision-making process on a well-established and scientific methodology will allow for more reasonable choices being made about costly solutions. Knowledge about HB-HTA may contribute to more justifiable tender decisions: “We, as an entity, wanted to participate in something new, innovative. During the project, not everyone knew what HB-HTA was. We wanted to learn about this methodology and improve the way we purchase equipment” (P5). It was also emphasized that the proper organization of data is a crucial aspect of making right decisions. The area of data analytics is increasingly influencing the decision-making process in medical facilities and the HB-HTA project further developed this skill: “Recently, our department has been expanded to include an analytical part, which will enable us to undertake all activities fully consciously. This was the crucial factor for us to participate in the project” (P2).

#### 3.1.2 Healthcare service valuation

One of the participants of the study wanted to start working towards shaping HB-HTA in Poland because the level of valuations prepared by the national Agency for Health Technology Assessment was repeatedly unsatisfactory and did not correspond to the actual costs that were generated by providing health services: “We wanted to implement this project because our institute has the appropriate capabilities to support the Polish HTA Agency in the process of preparing analyses and activities in the field of health technology assessment” (P3). Hospitals have faced the problem of too low valuation of medical services and the data obtained as a result of HB-HTA analyzes could be used to value services more appropriately.

#### 3.1.3 New technologies evaluation

The motivation for participating in the study mentioned by one of the hospital representatives was the possibility to gain a tool for assessing the implemented solutions: “There was a need to develop a way to evaluate new technologies and we also needed an appropriate methodology for that” (P3). The HB-HTA methodology would allow for the systematization of activities related to the implementation of innovations in hospitals.

### 3.2 Methods of assessing health technologies before the project

#### 3.2.1 Internal analyses and IOWISZ, external institutions

All respondents who took part in the study indicated that previously, short analyses had been carried out in their institutions, with documents prepared only for internal use. They also completed external applications under IOWISZ. At the same time, they indicated that the current solutions, such as IOWISZ, are not ideal. The assessment of introduced technologies is conducted in an *ad hoc* manner. It should be noted that the IOWISZ methodology is not solely concerned with the evaluation of medical technologies and does not directly inform decision-making processes in the same way that the HB-HTA does: “Health technologies had not been assessed in an informed way before” (P3). Previous activities in the area of technology assessment were informal, without specific rules and procedures: “I think that such a question [about internal analyses before the project] could probably be answered comprehensively by the management, although I know that previously such analyses had been carried out internally” (P4). Respondents indicated that the role of IOWISZ in the process of assessing solutions implemented in hospitals was not appropriate: “The only thing was submitting applications under IOWISZ, i.e., as part of the assessment of the advisability of the investment” (P3). Otherwise, IOWISZ is not binding at the decision-making level and it can cause inconsistency: “IOWISZ is not that simple—there are some decision points in IOWISZ that do not influence decision making. There is a lack of consistency in the assessment under IOWISZ, for example the National Health Fund may give a negative opinion, and the decision will still be positive at the political level. It does not make sense” (P3). HB-HTA did not develop in the post-project phase either: “The team has been formed, but at the moment we do not conduct any procedures in accordance with the HB-HTA methodology. Our responsibility is preparing assessments in a traditional way without HB-HTA methodology” (P5).

### 3.3 Benefits resulting from participation in the study

#### 3.3.1 Systematic way to analyse medical technologies

The experiences that respondents gained by participating in the HB-HTA project included becoming acquainted with articles from medical scientific databases and comparing technologies from different hospitals. Moreover, it should be emphasized that the respondents work, among others, in financial departments and have never dealt with the analysis of clinical trials. Due to the project the interviewees became acquainted with techniques such as PICOS. Organizational issues that are missing in reviews of clinical trials are also important for hospital employees: “We analyzed both the clinical, research, and organizational issues at the level of our hospital. The creation of systematic reviews occurs in research aspects of clinical trials, but the reviews are not used for organizational issues related to, among other things, work organization” (P1). Hospital representatives pointed out that proper data analysis was a very important issue and they can develop their skills by the project: “We learned how to use the acquired data appropriately” (P2). There is also a need to systematize activities in the assessment of modern medical technologies. Interviewees were content that the project provided them with structured methodology in HB-HTA reports”. “The analyst team developed methodological tools to use for preparing accurate assessment of medical technologies” (P3). Thanks to the workshops and the project, it was also possible to exchange experiences between hospitals: “Another advantage was contact with people from other hospitals and investment and design departments” (P4). Previously, teams in hospitals did not deal with health technology assessment in such detail and they did not use scientific databases in their daily work: “The HB-HTA methodology includes not only financial analysis, but also other comprehensive activities, such as analyses of articles from the Pubmed database. We were not guided by medical reasons and the people responsible for financial analysis did not search for materials in Pubmed” (P5). The respondents also pointed out that HB-HTA made it possible to look at the purchase of technological solutions not only from the perspective of the equipment costs, but also from the perspective of the processes of implementing a solution. Another respondent indicated that HB-HTA provides a new perspective on medical technology assessment, resulting from a literature review or analysis of the target group: “An important experience was certainly learning to take into account the costs of not only the purchase of technology, or any investment in technology, but also bearing in mind all the costs associated with the organization, coordination, and management of various types of projects. We approached HB-HTA from the perspective of the entire procedural change of a given project” (P1). HB-HTA allows to look at technologies from different perspectives.

### 3.4 Organizational and financial changes to implement HB-HTA in hospitals and the healthcare system

#### 3.4.1 Financial factor

In their statements, respondents indicated that HB-HTA requires financial resources and appropriate interest from the various healthcare institutions that would help disseminate this idea. Currently, no hospital in Poland fully benefits from the use of HB-HTA, and hospitals continuously need to ensure that project teams are not disbanded: “In my opinion, implementing HB-HTA takes both time and money. We have already prepared the methodology, but we need to be more aware of it” (P3). Lack of financial stability for HB-HTA teams could cause failures and pitfalls. Financing is a key issue to develop HB-HTA in Poland: “However, one of the main problems that resulted in the lack of implementation of HB-HTA in Poland is the lack of financing for HB-HTA teams” (P5). Hospitals will need more visible effects of HB-HTA and stability of employment of people responsible for HB-HTA in hospitals: “When the entity does not see any benefits, but rather the problems that pile up, it is difficult to convince it to use such a tool” (P1). Facilities are struggling with the problem of retaining employees, which may affect further activities towards HB-HTA; “The primary condition is to maintain the team that worked on the project” (P3).

#### 3.4.2 New standards in data processing

Respondents of the study highlighted the problem of data exchange between institutions, which often relies on very archaic ways of gathering information: “So, if we do not improve the standard of data exchange between stakeholders, this will only cause discouragement and simply a waste of time on data processing that is not necessary. This is a waste of time, often of very expensive or very busy specialists. Returning to the aspect of data exchange, the exchange of data on implemented activities is certainly necessary. Currently, it is not possible to exchange data between units because we do not have a platform for exchanging information” (P1). A poor level of information exchange may lead to the deterioration of the functioning of medical facilities. Appropriate steps towards data analysis are needed. Moreover, HB-HTA requires adequate resources, which must be provided by hospitals in the form of appropriate staff and standards: “There is no additional financing for this, so there is little interest in preparing this type of document. Such activities require time and commitment” (P5).

### 3.5 The need to modify HB-HTA methodology

#### 3.5.1 HB-HTA as difficult methodology

Respondents participating in the study highlighted that HB-HTA methodology is very extensive. One of the facilities shortened it to suit its needs. However, there are also respondents who believe that the entire parts of the methodology cannot be removed, since it would reduce the chances of drawing conclusions from the HB-HTA report. Nevertheless, the majority of interviewees pointed out that some individual parts of the report were too long and repetitive. Preparing or reading HB-HTA reports is too time-consuming: “HTA reports of some drugs are often very broad studies, several hundred pages long. Even if the HB-HTA report were 30 pages long, it would be difficult for the hospital director to read it. The directors of medical entities simply do not have time for that. This could be shortened in an internal document of a summary. The director should get the one-page summary” (P4). “The one drawback of HB-HTA methodology that could be improved is the fact that many actions were repetitive. The component parts of the methodology themselves were well describe and organized, but some of them were too often repeated in the report” (P5). “Creating the full HB-HTA report, even for such a large facility as ours, is a difficult task. We have retained the methodology, but we have significantly simplified the method of description and argumentation” (P1). “HB-HTA was sent directly to the management team. However, the director needs support from analysts because he does not have time to read 20-page documents. We need to provide information on how to decide if we should implement a given technology. Personally, I think this report is very broad. Preparing a literature review is a very difficult task. It could be shortened to a description of 2–3 sentences by describing the benefits of using a given solution” (P4). Shortening and simplifying the HB-HTA documents might be an incentive for more medical entities to employ this methodology, leading to more effective medical technologies implementation.

Based on the interviews, respondents noticed a number of advantages of implementing HB-HTA in hospitals. In their statements, they point to the need to systematize the approach to assessed medical technologies and the use of scientific resources (e.g., literature) and HB-HTA methodology might be an answer to that. On the side of barriers, they emphasized especially two aspects: the lack of willingness to finance HB-HTA and too extensive methodology that may be difficult to understand both for hospital employees and management staff. All benefits and barriers are presented in Table 3.

**Table 3:** Benefits and barriers related to HB-HTA implementation.

Benefits of introducing HB-HTA	Barriers related to the introduction of HB-_HTA
Basing decisions regarding the introduction of innovations on a specific methodology	The need to expand knowledge about the HB-HTA methodology
Introduction of EBM principles to evaluate solutions implemented in hospitals	The need to increase the number of people working in hospitals familiar with the HB-HTA methodology
Deepening the analyzes related to the implemented solutions	Lack of interest in HB-HTA on the part of management staff
Introduction of more advanced, scientific methods for assessing the cost-effectiveness of implemented innovations (e.g., literature review, PICO(S) analysis)	Too extensive a methodology that will not be understandable to the management staff or the report creators themselves
Making decisions about including health services in the benefit package	Lack of additional HB-HTA funding to develop such teams at the hospital level
Determining the actual benefits (both financial and organizational) from the implemented solutions	A problem with the quality of data shared between individual institutions in Polish healthcare
Formalizing the health technology assessment process at the hospital level	
Increasing interest in advanced medical technologies	

## 4 Discussion

The facilities that participated in the study indicated that the HB-HTA methodology enables systematization of knowledge and basing facility managers’ decisions on specific data. Hospital representatives emphasized that the HB-HTA teams formed as part of the pilot study are still functioning and internally assessing the technologies introduced to the units. The guidelines for creating HB-HTA reports themselves may, in the opinion of hospital employees, be slightly modified towards a business analysis rather than a scientific one. Facilities also need to derive significant benefits from HB-HTA to be willing to employ it. Health technology assessment at the hospital level should also be supported by central institutions (the Ministry of Health, the National Health Fund) in order to develop.

Many interviewees also referred in their statements to activities related to the Instrument for the Assessment of Investment Applications in the Health Sector (IOWISZ). IOWISZ was introduced to organize healthcare investments and develop an appropriate method for allocating public funds. Hospital investment activities are subject to assessment by the voivode or the competent health minister. The usefulness of IOWISZ, as well as its issued opinions on the advisability of a given investment, is limited by the source of information (22). The respondents participating in the study pointed to drawbacks resulting from the chaos surrounding how IOWISZ functions. It has weaknesses in how it assists in making investment decisions, as well as in the fact that it is not fully binding, as the final decision on commencing activities may result from political factors. Similar indications have been presented by other experts who point out that IOWISZ does not increase the effectiveness of public spending. The respondents emphasized that organizational activities should resolve clinical issues through the implementation of HB-HTA because the effectiveness of a given therapy, its safety, profitability, and impact on the hospital budget should also be taken into account. The Hospital Health Technology Assessment should determine the appropriateness of the investment in line with the literature and based on up-to-date data. The respondents indicated that making decisions based on specific principles is a primary benefit of HB-HTA.

HB-HTA has been developed not only in Poland, but also as part of the AdHopHTA project, in which experiences were exchanged regarding the forms of health technology assessment at the hospital level in nine selected European countries (Austria, Denmark, Estonia, Finland, Norway, Italy, Spain, Switzerland, and Turkey) (23). The aim of this project was to indicate the role of HB-HTA in shaping innovative medical technologies, as well as identifying good practices for HB-HTA methodology. The Hospital Technology Assessment is intended to fulfill the following tasks:

- Properly assessing implementation time and adjusting the pace of introducing modern technologies to hospital budgets;
- Providing appropriate information about a given technology to representatives of the hospital management;
- Increasing the efficiency of using technology in hospitals;
- Improving patient safety (22).

The project also defined the stages of adopting medical technology in hospitals. Here are the most important activities in implementing innovations in hospitals:

1. Initial clinical analysis.
2. Determination of the price and impact of technology and definition of tender requirements.
3. Market analysis and consultations.
4. Selection of the tender procedure.
5. Analysis of offers and issuing a final decision.
6. Logistics and tenders (22).

Evidently, the AdHopHTA project raised not only purely scientific issues, but also those of organization and documentation. The respondents expressed a need to base HB-HTA reports more on business aspects, and to a lesser extent on clinical aspects or advanced scientific literature. They also indicated that the methodology created by the project team should be simplified. The activities of hospital employees are multidimensional, and the work of facility directors requires familiarizing themselves with many pieces of information at once. They also postulated that there should be further training of the current staff and that new people should be employed to prepare such analyses. Lazarski University, which was the first in Poland to organize postgraduate studies in the field of HB-HTA, is trying to fill the market niche in this area. Creating such a group of specialists would increase the professionalization of health technology assessment at the national level, and graduates of these studies could become a human resource for hospital facilities as well as institutions such as AOTMiT or the National Health Fund (23).

The very concept of professionalizing the implementation of innovative medical technologies results from, among other factors, the fact that the Polish healthcare system is increasingly focusing on quality. In 2023, the Act on Quality in Healthcare came into force (24). Basing decisions on an HB-HTA report could be important for improving management techniques in medical facilities. The introduction of HB-HTA does not need to be constrained only to a financial dimension, e.g., increasing the funds allocated to units that use HB-HTA. As part of HB-HTA methodology, one can also take into account the perspective of pro-quality indicators derived from the management sphere, which, through their measurability, can help in the implementation of health services. The quality of hospital activities may be improved by reorganizing department activities or adjusting the amount of staff. Increasing quality in healthcare will require appropriate accreditation of hospital units providing services financed from public funds. HB-HTA could be an important aspect of improving the assessment of the advisability of investments for some hospital facilities, especially highly referenced hospitals.

It is hopeful that the hospitals that participated in the pilot (e.g., the University Clinical Hospital in Gdańsk etc.) have been promoting good practices in this area. Their maturity in HB-HTA analyses could make them role models for other entities. Evaluating innovative technologies based on HB-HTA methodology provides these hospitals with many benefits; above all, HB-HTA allows them to use effective, safe, and cost-effective innovations, which translates into good clinical and economic results. Additionally, the Act on Quality and Patient Safety puts pressure on institutions to implement tools that optimize clinical, consumer, and management activities. Practice shows that better results are achieved by entities managed on the basis of data and critical analyses of the cost effectiveness of their investments.

This study indicates the direction in which HB-HTA should be developed, based on interviews with selected representatives from hospitals participating in the HB-HTA pilot. The assembled team of experts demonstrated significant knowledge of HB-HTA, not only from the perspective of the hospital, but also from the perspective of the entire healthcare system. Hospital employees participated in both the HB-HTA project and the report preparation stage. The information provided indicates that hospitals are where most development of HB-HTA occurs because, due to such activities, implementing solutions in hospitals can become as professional as the majority of research work. Also, this study unavoidably has some limitations. The research was conducted only on the small group of respondents who took part in the study. Conducting a largescale investigation could address the research problem more effectively and be a valuable source of information on the implementation and further development of Hospital Health Technology Assessment in Poland. Other stakeholders responsible for medical innovations can be taken into account (from Ministry of Health, National Health Fund, Polish HTA Agency). However, it should be emphasized that approximately 70% of the hospitals participating in the pilot phase of the project were surveyed and that the interviewees were highly qualified and understood the content of the questions asked. Moreover, they often initiated broadening of the issues discussed, e.g., regarding how other healthcare entities function in the business environment of hospitals. More interviewees could make the analysis by showing the perspective from various healthcare institutions. However, the aim of the study was to getting to know the opinion of hospital representatives on the development of HB-HTA in their facilities.

HB-HTA has been implemented in several European countries to assess hospital investments, and these experiences were presented as part of the AdHopHTA project. One such country is Finland, where hospital districts and the Finnish Agency for Health Technology Assessment, FinoHTA, were invited to participate in the development of HB-HTA. As part of their cooperation, these institutions have created a program for process management of medical solutions. The University Hospital in Helsinki was the first to carry out mini-HTA reports in accordance with this program, taking into account HTA reports in terms of their usefulness for hospital units (25).

A study of the development of HB-HTA in France showed that hospitals use this methodology primarily for informational purposes in making decisions about implementing a given innovation. French hospitals have special procedures for implementing solutions, especially regarding cost analysis. Clinical hospitals feature the most developed level of HB-HTA because it is there that the rigor of assessing medical technologies in accordance with HTA is most visible. Therefore, the French experience is consistent with the fact that in the Polish project, HB-HTA reports were prepared only by hospitals with a high reference level (26).

The process of formalizing health technology assessment has been divided into four stages in the literature:

- An independent unit, in which HB-HTA is developed through unofficial contacts, and the willingness to evaluate the implementation of solutions is mainly demonstrated by clinicians who are ambassadors for changes in medical facilities. In this solution, there is no cooperation with any other healthcare institution, and the mini-HTA report is primarily used internally for hospital managers;
- A stand-alone unit, in which cooperation takes place with external healthcare actors to a significant extent, with a team of highly qualified specialists working inside the hospital. This is the most common HB-HTA model in Europe;
- An integrated unit, in which a few specialists work, including some from units from outside the hospital. Cooperation with HTA agencies is informal;
- An integrated specialist unit that formally cooperates with national HTA agencies. HB-HTA is the work of not only hospital staff, but national and regional HTA agencies are also involved in HB-HTA (17).

In Poland, the first HB-HTA model has the greatest chances of developing. Looking at the results of the study conducted by the research team, Polish healthcare institutions are not particularly interested in developing HB-HTA and support for hospitals. The HB-HTA model established in the project indicated the roles of many actors related to the healthcare system in shaping individual tasks. The coordinating role at the national level in the model was to be played by the Ministry of Health; compliance of reports with HTA methodology was to be implemented by AOTMiT; and the National Health Fund would create financing paths for innovative health services based on HB-HTA. Unfortunately, this form of HB-HTA, with many entities simultaneously fulfilling different roles, was not successful due to certain activities not being carried out. For example, the Ministry of Health has not implemented regulations supporting the development of health technology assessment in hospitals. Neither has the national payer offered any financial incentives to hospitals that implement HB-HTA.

## 5 Conclusion

Hospital Health Technology Assessment in Poland at the hospital level still requires many activities and increased interest from various healthcare institutions. Employees of the hospitals participating in the HB-HTA project have implemented investment assessment methodology, although it is more applicable to internal activities and has been adapted to the context of the given facility. There are no appropriate incentives for using HB-HTA methodology, either financially or organizationally. Such activities would make assessing the implementation of modern technologies more widespread and systematic.

Based on the experiences of the interview participants, it can be concluded that HB-HTA needs strong institutional support in terms of implementing activities, both organizationally and economically. In order to create HB-HTA units, the will of various health care participants is needed that such units are needed in hospitals. Before health technology assessment teams are created at the hospital level, funding for training, determining the shape of the HB-HTA team and understanding from the management staff are needed. For this to happen, further development of activity towards HB-HTA is necessary.

## Data Availability

The original contributions presented in the study are included in the article/supplementary material, further inquiries can be directed to the corresponding author.
