# Supplementary material for: Implementation and development of hospital-based health technology assessment in Poland from the perspective of hospital representatives: qualitative research
Source: Front Public Health. 2024 Oct 8;12:1426420. doi: 10.3389/fpubh.2024.1426420 (PMC11493587; doi:10.3389/fpubh.2024.1426420)
Supplement: Supplementary file 1 [file Data_Sheet_1.PDF]

MF: How did you find out about the HB-HTA project?
MG: I'm not sure how exactly the hospital found out about the project, but the HB-HTA team was formed by contemporary director, who was responsible for coordination and development. He gathered data and set up the team consisting of the head of the Management Accounting Department, the head of the Financial Planning and Analysis Section (me), and one person from the Coordination Department. We came from three different fields, which allowed us to combine our experiences.
MF: How did you assess the implementation of some innovative technologies in your facility before this project?
MG: HB-HTA does not function within the hospital in the form in which it was presented in the design phase of the project. The team has been formed, but at the moment we do not conduct any procedures in accordance with the HB-HTA methodology. We do not write HB-HTA reports, although this is planned. We are writing sample papers so that we can present them to the management and demonstrate what such an assessment might look like. There is no additional financing for this, so there is little interest in preparing this type of document. Such activities require time and commitment. Moreover, these are not the issues that can be easily dealt with during working hours, especially since the team consists of experts from various fields, so it would require work after hours. Our responsibility is preparing assessments in a traditional way without HB-HTA methodology. Heads of hospital wards are responsible for providing information on why this equipment is necessary from a substantive perspective. We, as the Financial Planning and Analysis Section, create financial analyses for those purchases. Each department deals with its own area. There is no team that analyzes everything comprehensively. It is the director who makes the final decision based on substantive evidence from the substantive units.
MF: What prompted you to participate in this project?
MG: It's hard for me to say, because you would have to ask the director about it. We, as an entity, wanted to participate in something new, innovative. During the project, not everyone knew what HB-HTA was. We wanted to learn about this methodology and improve the way we purchase equipment. We have a lot of equipment that could be analyzed as part of HB-HTA due to the fact that the new headquarters of the university hospital has just been built. However, we still need to buy innovations. The HB-HTA project was compatible with our activities, so from our perspective it was interesting.

MF: Can you describe your experiences connected with preparing this report? What new skills have you gained thanks to this project? What were the benefits of participating in the project for the facility?

MG: The HB-HTA methodology includes not only financial analysis, but also other comprehensive activities, such as analyses of articles from the Pubmed database. Previously, financial units were not responsible for this, we were not guided by medical reasons and people responsible for financial analysis did not search for materials in Pubmed. The HB-HTA report allowed for a comprehensive description of a given medical technology. We had a full review: medical review, as well as organizational and economic part. The director has a very good tool to make informed decisions, because all of the data is gathered in one place.

MF: Did any external institution, such as HTA Agency or NHF, help in preparing the report?

MG: We did not use HTA Agency or NHF data. We tried to contact HTA Agency, but we did not receive any data we needed from there. Our work was too innovative for HTA Agency to help us as it concerned the IRR recorder.

Not so long ago, HTA Agency started collecting stroke data from us because they needed to see what the National Health Fund's valuations look like. Thanks to the fact that the data will come straight from our facility, the valuation of the service will be at the appropriate level. We really liked this project since we could take into account the allocation of equipment in a certain geographical area, as in IOWISZ, so as not to buy too much of something and assess if it is really worth investing in something.

MF: Why has HB-HTA not been fully implemented in Poland?

MG: In my opinion the idea of HB-HTA itself is fine. However, one of the main problems that resulted in the lack of implementation of HB-HTA in Poland is the lack of financing for HB-HTA teams. A facility such as ours would have to use its own resources to establish such a team. It should also be indicated what benefits establishing such a team would bring to the institution. Such a benefit for the entire healthcare system could be the effective spending of public funds. Support in creating HB-HTA could be provided by the National Health Fund. Institutional support is needed at the level of other healthcare actors.

We have an example from Gdańsk, where the hospital team started creating mini-HTA reports. They reduced this methodology a bit. HB-HTA team certainly needs to come from within the hospital to be able to interpret the data well. It must be embedded in the operating conditions of the hospital.

The analyzes we carry out are not similar to what was in the project, because they are simplified. We have a path to investment acceptance. It is not called HB-HTA.

MF: Should the entity preparing HB-HTA reports receive additional money for implementing this type of solution and for assessing a given technology before it enters the hospital practice?

MG: Financial issues probably contributed to the fact that HB-HTA did not take off as much as we thought it would. There must be a visible benefit for this institution introducing HB-HTA. A benefit for the hospital related to the appropriateness of purchasing the equipment. HB-HTA can also help hospitals to stop purchasing equipment that will not be in demand. As a hospital, we have a lot of innovative equipment. As employees of the Financial Planning and Analysis Department, we also have a very heavy workload, and we aren’t able to work in such a team, because we simply do not have time. We should either change the way we work or hire new people.

MF: And when it comes to the actors, who would shape this institutional support?

MG: It's hard for me to say, but the National Health Fund and the Minister of Health could play such roles, which could mean that an entity that has HB-HTA could receive a larger lump sum. There should be a higher lump sum for this additional form of activities. Similarly, we as a unit receive a larger lump sum thanks to the fact that we have a certificate in microbiology. The change of director did not help HB-HTA either. HB-HTA is not standardized in our country, we need a larger team.

MF: Is there a need to modify project documents and design recommendations?

MG: I am not sure whether this methodology should be modified as it is very comprehensive. In HB-HTA, the procedures are transparent. Simplifying any part may make the assessment

incomplete and somewhat instinctive. If the methodology were to be simplified, it would be in such a way as to retain the most important elements of this assessment without removing any part of the analysis, such as organizational or financial analysis. If we do it in a haphazard manner, there may be a problem with drawing conclusions from such reports.

Writing the report for our team was quite easy as we divided our tasks. The Purchasing Department manager was familiar with Pubmed database, so he dealt with the analysis of literature. The Coordination Department manager was responsible for establishing if the hospital is able to implement HB-HTA from the organizational point of view and was also liable for contact with medical staff. We, as the Financial Department, dealt with financial analysis. A significant advantage of our team was that each of us knew English. I think that people working in such teams cannot be accidental and analysis cannot be done with some scheme, each one should be individual.

The one drawback of the HB-HTA methodology that could be improved is the fact that many actions were repetitive. The component parts of the methodology themselves were well described and organized, but some of them were too often repeated in the report.

HTA reports of some drugs are often very broad studies, several hundred pages long. Even if the HB-HTA report was 30 pages long, it would be difficult for the hospital director to read it. The directors of medical entities simply don't have time for that. It could be shortened in an internal document of a summary. The director should get the one-page summary.

As I said before, currently, the HB-HTA methodology is quite long, and many elements are repeated. At the same time, we should not shorten entire fragments, such as a clinical analysis, which are valuable and hadn’t been taken into account before by us.
