# Supplementary material for: Implementation and development of hospital-based health technology assessment in Poland from the perspective of hospital representatives: qualitative research
Source: Front Public Health. 2024 Oct 8;12:1426420. doi: 10.3389/fpubh.2024.1426420 (PMC11493587; doi:10.3389/fpubh.2024.1426420)
Supplement: Supplementary file 2 [file Data_Sheet_2.PDF]

MF : How did you find out about the project?

AS : Let me start by saying that I work in the organizational department, which is partly responsible for writing various projects, mainly the investment ones, but we also prepare offers of different medical competitions. On the GrupaBiznes website I found a document with invitation to the HB-HTA pilot project and our director, Prof. Ptaszynski, also got interested in the project. Therefore, we immediately started thinking about participating in the project due to his involvement. Prof. Ptaszynski participated in the project from its initial stage.

MF: Before this project, had you assessed any medical innovations in accordance with some previously established internal methodology?

AS: I think that such a question could probably be answered comprehensively by the management, although I know that previously such analyses had been carried out internally. When it comes to innovations, discussions are most often held at the management level. Applications usually come from interested parties (e.g. clinicians).

MF: Why were you interested in this project?

AS: I am not sure what guided the Director before making the decision to participate in the project. We are an innovative institution, and we place great emphasis on that field. We try to take part in multiple projects. Personally, I wanted to learn something new about hospital cost management. I also wanted to find out how data from other hospitals is collected. Those factors had a big impact on my participation in this project.

MF: What was your experience in writing the HB-HTA report? Who managed the tasks at the level of your facility?

AS: The Director did not participate directly in writing and collecting the data, but performed a management function. I work in the organizational department. I am involved in raising funds for the hospital, but only from the investment point of view, i.e. purchasing. We received assistance from the worker who is responsible for contracts in our hospital. The Director of our library supported us in our activities, too. He dealt with the issue of the literature review that was essential for us. There were about 3-4 people in our team.

MF: Have you had contact with any external entity?

AS: No, we haven’t.

MF: What new skills did you gain from the participation in this project?

AS: The knowledge gained during the project was useful for writing applications within IOWISZ. Another advantage were contacts with people from other hospitals and investment and design departments. Thanks to this, we learned how they collect data. We also learned how HB-HTA can develop in Poland and we can see how other hospitals deal with that.

MF: What support should be provided by systemic institutions?

AS: The core issue is the awareness of the staff that such assessment is needed. We know they are focused on their work, but we also need their help with access to data. We met resistance on their part. Moreover, I believe that this report was very time-consuming and difficult to prepare. You need to dig deeper into HB-HTA to understand it. The people who prepare this report usually have no medical knowledge. We wrote about gamma cameras. We had to learn how to provide this service, which was very time-consuming. I believe that there should be a dedicated team dealing with HB-HTA in hospitals, but the team should be properly trained and prepared in advance. It should also be familiar with preparing a literature review. Such a review could also be performed by an external company. People working in our department do not have much knowledge about literature reviews.

MF: Systematic reviews could be done by higher education facilities.

MF: Should the project materials be slimmned down?

AS: Yes, materials should definitely be slimmed down. HB-HTA was sent directly to the management team. However, the Director needs support from analysts because he does not have time to read 20-page documents. The most important thing is the summary. We need to provide information on how to decide to implement a given technology. Personally, I think this report is very broad. Preparing a literature review is a very difficult topic. It could be shortened to a description of 2-3 sentences by describing the benefits of using a given solution. We also have a problem with IOWISZ, as it is often difficult to adapt the criteria to the investment. We recently analyzed the issue of thermal modernization of public utility facilities in IOWISZ. I think that a solution such as HB-HTA or IOWISZ can be used, for example, when purchasing a new MRI.

MF: Should the science component be reduced to a business component?

AS: Yes, definitely. Additionally, I would like to add that an important aspect is the cost perspective for the hospital. Cost-benefit analysis. The number of people admitted per month

should be analyzed and, if necessary, the limit of services provided should be increased. The economic analyses were most valuable.

As for the form of conducting the courses, there were very few practical classes, there was a lot of theoretical work and little practical work. Only after the entire training program, when writing the report itself, did we learn how to prepare it.

MF: Thank you for the interview.
