# Supplementary material for: Implementation and development of hospital-based health technology assessment in Poland from the perspective of hospital representatives: qualitative research
Source: Front Public Health. 2024 Oct 8;12:1426420. doi: 10.3389/fpubh.2024.1426420 (PMC11493587; doi:10.3389/fpubh.2024.1426420)
Supplement: Supplementary file 3 [file Data_Sheet_3.PDF]

MF: Our conversation will be transcribed using MS Teams. My first question is: How did you find out about the HB-HTA project?

TB: We were the authors of the idea itself, so this question does not directly apply to us. We did not learn about HB-HTA either, because we designed that concept.

MF: Have you had any experience with assessing highly advanced technologies in your own facility before taking part in the project? Have you dealt with HB-HTA before?

TB: No, we haven't. Our activities began with the contact with the Ministry of Development. Our project was a part of the so-called "fast track" to get a grant. I collaborated in this area with prof. Piotr Szymański (Scientific Director of the Institute). We wanted to implement this project, because our institute has the appropriate capabilities to support Polish HTA Agency in the process of preparing analyses and activities in the field of health technology assessment. We would like to take part in calculating the value of health services as we have got relevant data. The valuations prepared by National HTA Agency are nationwide. Therefore, from our perspective, they are inaccurate and simplified. The costs are average. Much information is missing, e.g. how much a given service actually costs. During the talks with the Ministry we pointed out that the costs of some specific entities are completely different than the costs they average.

MF: Had you assessed health technologies before?

TB: Health technologies had not been assessed in an informed way before. There was no methodology, everything was done adhoc. We wanted to systematize this in our country.

MF: Who in your department was responsible for preparing the report? Was it a group of people or rather one specific person?

TB: The research team consisted of a group of analysts. There were also people with economic education, they currently work in various departments of our institute. The team continues to function on the basis of the director's decision. The same team is responsible for cooperation between the National Health Fund and the Ministry of Health.

MF: Did you cooperate with HTA Agency on the preparation of this report?

TB: We did not share any data with Polish HTA Agency in the report. We did not cooperate with any external entity either. The report was prepared solely by us.

MF: What new skills did you develop while preparing this report?

TB: Thanks to the structured methodology the analyst team developed methodological tools to use for preparing accurate assessment of medical technologies. I think that was the most important thing. We could work in a more systematized way.

MF: What organizational and financial conditions must be accomplished to develop HB-HTA?
TB: The primary condition is to maintain the team that worked on the project. We perform tasks related to the National Cardiology Network and this year we would like to perform four tasks based on HB-HTA methodology.

Finances are another crucial factor. We have finances within the National Cardiology Network. Additionally, we choose technologies based on the National Cardiology Network because we have the resources to prepare such recommendations.

MF: Is HB-HTA needed in our healthcare system? Why has this solution not been implemented into the Polish healthcare system?

TB: In my opinion, implementing HB-HTA takes both time and money. We have already prepared the methodology, but we need to be more aware of it. Moreover, we need to have greater financial possibilities as a system. However, it should pe pointed out that it is impossible to implement HB-HTA everywhere (in every hospital), because it does not make much sense. We would like to have HB-HTA teams to see something that employees of HTA Agency or other institutions cannot see. HTA Agency in its calculations often relies on certain simplifications that are not adequate for the situation of a healthcare entity such as ours.

MF: Should the shape of these documents, including design documents, be changed?

TB: We should use the HB-HTA methodology as in PRINCE, i.e. we have a set of options, but we choose those that we need at the moment or those that we can use. I am not straigt-laced in terms of the shape of this methodology. It may change, for example at the government level, it may be the part of HTA Agency activities. As a lot of work is carried out by the analysts, the most crucial issue is their training so that they know what they do.

MF: Voivodeship Offices were supposed to deal with HB-HTA, but they were not able to do it in an appropriate manner.

TB: IOWISZ is not that simple - there are some decision points in IOWISZ that do not influence decision making. There is a lack of consistency in the assessment under IOWISZ, the National Health Fund may give a negative opinion, and the decision will still be positive at the political

level. It does not make sense. The system is inconsistent. Voivodeship Offices alone would not have the resources to seriously deal with HB-HTA. IOWISZ operates primarily in the Silesian Voivodeship. IOWISZ in other voivodeships is implemented at a low level. Political aspects are also important, because political bodies can help implement this change.

MF: Should HB-HTA methodology be modified?

TB: HB-HTA methodology must be constantly modified. People working on reports must be constantly trained. Also, facilities need financial resources to make HB-HTA viable, but it will only be done by entities with large budgets. Large entities are always partners, whether for the National Health Fund or the Ministry of Health, as a stakeholder for medical services evaluation.
