# Supplementary material for: Implementation and development of hospital-based health technology assessment in Poland from the perspective of hospital representatives: qualitative research
Source: Front Public Health. 2024 Oct 8;12:1426420. doi: 10.3389/fpubh.2024.1426420 (PMC11493587; doi:10.3389/fpubh.2024.1426420)
Supplement: Supplementary file 4 [file Data_Sheet_4.PDF]

MF: First of all, how did you as a facility learn about the HB-HTA project?

DB: I found the information on the Internet, but we also exchanged information between our director and the director of the Institute of Cardiology.

MF: Had you assessed such modern technologies before? How had you evaluated these technologies previously?

DB: No, we hadn't. The only thing was submitting applications under IOWISZ, i.e. as part of the assessment of the advisability of the investment. We performed more complex analyses of activities related to the building of the hospital wards or departments.

MF: What motivated you to participate in HB-HTA project?

DB: We are the department that deals specifically with various areas related to screening programs. Thanks to the participation in the project, management staff can make decisions based on data. Recently, our department has been expanded to analytical part, which will enable us to undertake all activities fully consciously. It was the crucial factor to participate in the project.

MF: What are your impressions as to preparing this report? What are your most important experiences in running the report?

DB: We realized that we need to approach each topic from different perspectives: from the literature, expert, financial and target group perspective. We became aware of the fact that we can approach topics related to medical technologies in a completely different way and that it also brings results.

MF: How many people worked on the project? Did any external institution help in preparing the report?

DB: We prepared the applications ourselves. Our team included me and two people from my department. Also the Director Zawadzki took part in the project. We acted based on our own data as well as data included in publicly available registers, e.g. the National Cancer Registry or the National Health Fund. We did not submit any applications, for example to other hospitals or associations, and we did not cooperate with any other entities or ask for additional data from them.

MF: What skills did you acquire during the project?

DB: We learnt how to use the acquired data appropriately.

MF: So you hadn't relied on scientific literature so often before in your internal reports?

DB: Previously no, we had only checked our internal data. Sometimes we had also checked the data in the National Cancer Registry, but we hadn't used analyses or information published in Pubmed or other similar sources.

MF: When it comes to organizational issues, what changes should be introduced in your facility in order to implement HB-HTA? What changes should be made in the entire healthcare system?

DB: We will have to implement internal arrangements at the level of hospital and to slightly change the decision-making path regarding investments in the scope of medical services. We need to use the HB-HTA methodology for investments activities. This area related to IOWISZ would have to be revolutionized in general, the transition to the HB-HTA methodology would constitute such a change. The methods of assessing investments in hospital facilities should be changed. The scope of services should also be systematized too, which would also increase patients' access to them.

MF: Why has HB-HTA not been fully implemented? Should HB-HTA methodology be changed?

DB: The IOWISZ system is not that bad. It has some drawbacks, including the lack of important elements at the application submission stage. There is no analysis of whether doctors will be available or how many patients will actually use a given medical service. In order to systematize and to implement certain investment areas in the country, we need to synchronize IOWISZ with HB-HTA. Changes in the medical field, in decision-making, and in the National Health Fund in terms of contracting would be necessary too.

MF: What institutions should support HB-HTA and to what extent?

DB: The Ministry of Health and the National Health Fund are responsible for covering medical services financially. These two organizations could help to implement HB-HTA. It would be necessary to rethink how to approach the entire healthcare system in a different way and how to implement these activities like HB-HTA.

MF: Should the HB-HTA methodology be based on more recent data?

DB: From a purely technical point of view, the National Health Fund or the Ministry of Health are in possession of up-to-date data based on the medical services they provide, which are reported to them and transferred once a month. Combining this with the HB-HTA methodology would be the appropriate course of action to update the information even more. We would have a real set of transparent data with the described methodology without ambitious influences from various medical entities. We would have real coordination based on real data collected and discussed in real time.

MF: What technologies have been evaluated in your facility?

DB: In our facility two technologies were evaluated, one related to changing the patient path for diagnostics in the treatment of lung cancer. We internally changed and optimized the paths to shorten the diagnostic time. As the second technology, we evaluated a virtual colposcope related to cervical diagnostics. At the moment, we have not found the funds to move forward with that project.
