# Supplementary material for: Implementation and development of hospital-based health technology assessment in Poland from the perspective of hospital representatives: qualitative research
Source: Front Public Health. 2024 Oct 8;12:1426420. doi: 10.3389/fpubh.2024.1426420 (PMC11493587; doi:10.3389/fpubh.2024.1426420)
Supplement: Supplementary file 5 [file Data_Sheet_5.PDF]

MF: How did you find out about the HB-HTA project? Why did you take part in the project?

DS: We were simply invited. I don't remember exactly who invited us (it was through Mrs. Małgorzata Gałązka-Sobotka or Mrs. Iga Lipska).

MF: Had you used this methodology before?

DS: No, we hadn't used it before. There was some cooperation with HTA Agency earlier, as well as internal project evaluations, but they did not have the structured, orderly form that was developed as part of the project, which we later adapted to our own needs.

MF: What prompted you to participate in this project?

DS: There was a need to develop a way to evaluate new technologies and we also needed an appropriate methodology for that.

MF: Can you describe your experiences connected with preparing this report?

DS: An important experience was certainly learning to take into account the costs of not only the purchase of technology or any investment in technology, but also bearing in mind all the costs associated with the organization, coordination, and managing various types of projects. New technology must not only mean the purchase of something (e.g. a device), but it may also be process change, e.g. in terms of employing new people or creating new job positions, or organizational change that may result in specific consequences. The reports we prepared as part of the project had a very strong process component. Among other things, we took into account the costs of the process and the impact of handling a given process. We put emphasis on clinical effects. We approached HB-HTA from the perspective of the entire process change that concerns a given project.

MF: How many people were involved in preparing such a report?

DS: We prepared two reports as a part of the project. First, there was a preliminary report while the project was being developed and it concerned bariatric coordinated care. This was even before the regulation regarding KOS-BAR (Coordinated Specialized Care in Bariatrics) was published since we knew that such a coordinated care solution may appear soon. The KOS-BAR consultations were still in the very early stages. We calculated and described it as part of

this methodology (HB-HTA) and in this way prepared for organizational changes. The report showed us very clearly what changes in the structure, staff employment and patient service flow are necessary for such a process to be successful. As a result, later we had a ready reference point when financing under KOS-BAR appeared.

MF: Have you also cooperated with any other entities? Would someone share the data with you?

DS: We did it on our own and possibly used literature data. There are no entities sharing medical data in Poland. I am not aware of any entity that can make medical data available in a sensible way, apart from, of course, some aspects that are available through the National Health Fund in the context of the so-called “healthy data” or something. This is very fragmentary data and, in addition, it is made available with a considerable delay, so it is often not suitable for the current situation. The second report we prepared was also a process report and it regarded coordinated care under COPD. It was also based on our experiences and the report helped to systematize it all. It allowed us to describe and also to determine and count the costs of introducing coordinated care in COPD.

MF: What have you learned during the project? What skills have you acquired?

DS: We have learnt a systematic approach to literature, a critical way of selecting articles, i.e. creating an entire clinical analysis, creating a PICOS analysis as part of the preparation of input data. We analyzed both the clinical, research and organizational issues at the level of our hospital. The creation of systematic reviews occurs in research aspects of clinical trials, but the reviews are not used for organizational issues related to, among others, work organization.

MF: In your opinion, what should be the conditions for the effective implementation of HB-HTA for your facility, when it comes to issues of organizational and financial conditions within the organization? What should change on the part of other stakeholders of the system, such as the National Health Fund, AOTMiT or the Ministry of Health?

DS: What is crucial is having some standardization of data, defining how data is exchanged and better communication during data transfer. I must point out the terribly formatted table on the Ministry of Health's website. We have to do a lot of strange things in Excel so that the data can be sorted. We suffer to make documents look nice, not to make them useful. From the technical point of view, there is simply no standard for data exchange. The same is true between AOTMiT and units.

Data exchange is also carried out in a scandalously anarchic way. Comarch, which operates the AOTMiT platform, was asked why the system is so archaic and as an answer it provided a list of several dozen reasons why this cannot be changed. So, if we do not improve the standard of data exchange between stakeholders, it will only cause discouragement and simply a waste of time on data processing that is not necessary. This is a waste of time, often of very expensive or very busy specialists.

We do not use HB-HTA in the same formula as in the project. After the project, we developed an internal method, simplifying the procedure to suit our needs. We develop a document that contains all the elements of this report and also takes into account internal factors and quality components. As part of the project itself, we did not emphasize this aspect, we did not even notice it. Qualitative measures of the unit's work. In our internal HB-HTA, we have a large part that qualitatively assesses certain measures of the work of a given unit that applies for technologies.

Returning to the aspect of data exchange, the exchange of data on implemented activities is certainly necessary. Currently, it is not possible to exchange data between units because we do not have a platform for exchanging information. For example, in the case of an oncology network, it is almost impossible to obtain information that a patient is continuing treatment in another unit. This information can only be obtained with the P1 platform, but it concerns individual patients, and we want to study the behavior of groups of patients and quantify it. We do not have such mechanisms at all, we do not even have a mechanism to support such a process. Currently, data is exchanged through individual contacts or more or less accurate estimates are made.

MF: Why has not HB-HTA been fully implemented to Polish healthcare system?

If we want to implement a tool, e.g. HB-HTA, it must bring some benefits to the entity. When the entity does not see any benefits, but rather the problems that pile up, it is difficult to convince it to use such a tool. I don't mean just the financial benefits, but such a benefit may be for example the ability to optimize processes. But currently employing HB-HTA does not result in easier access to selected technologies.

MF: How external institutions could support HB-HTA? Is HB-HTA needed in our healthcare system?

DS: The activity of external entities should focus on integrating and comparing the data gathered as a result of analyses and showing their outcomes. It would be the most advisable.

If an entity is able to perform an HB-HTA assessment of a given process, this technology can be implemented much better in terms of quality. HB-HTA also requires indicators of the effect and specifying how this effect will be measured, both clinically and economically. This, in turn, easily leads to the creation of records of operational procedures and quality measures of these procedures. The result is an improvement in the quality of services, as well as the ability to predict, measure and control them. These are specific effects for the system that may later have some financial measure. I also think that access to pilot studies could be easier.

MF: Is there a need to modify project documents and design recommendations?

DS: Creating the full HB-HTA report, even for such a large facility as ours, is a difficult task. For us, this is a tedious, difficult, time-consuming and labor-intensive task. We have retained the methodology, but we have significantly simplified the method of description and argumentation. Thanks to this, we are able to conduct such an assessment together with clinicians during several online meetings in approximately 2-3 weeks. This assessment is quick, but still reliable. In addition, we have expanded this report to include the element of qualitative assessment of the process of the entity requesting it.
